# Why Does COVID-19 Affect Patients with Spinal Cord Injury Milder? A Case-Control Study: Results from Two Observational Cohorts

**DOI:** 10.3390/jpm10040182

**Published:** 2020-10-21

**Authors:** Enrique Calvo, Nerea Corbacho-Alonso, Tamara Sastre-Oliva, Estefania Nuñez, Patricia Baena-Galan, German Hernandez-Fernandez, Miguel Rodriguez-Cola, Irena Jimenez-Velasco, Fernando J. Corrales, Claudia Gambarrutta-Malfati, Francisco Gutierrez-Henares, Elisa Lopez-Dolado, Angel Gil-Agudo, Jesus Vazquez, Laura Mourino-Alvarez, Maria G. Barderas

**Affiliations:** 1 Proteomics Unit, Centro Nacional de Investigaciones Cardiovasculares (CNIC), 28029 Madrid, Spain; ecalvo@cnic.es (E.C.); estefania.nunez@cnic.es (E.N.); patricia.baena@cnic.es (P.B.-G.); 2 Cardiovascular Proteomics Laboratory and CIBER-CV, CNIC, 28029 Madrid, Spain; 3 Department of Vascular Physiopathology, Hospital Nacional de Paraplejicos (HNP), SESCAM, 45071 Toledo, Spain; ncorbacho@sescam.jccm.es (N.C.-A.); tsastre@sescam.jccm.es (T.S.-O.); ghernandezf@externas.sescam.jccm.es (G.H.-F.); lmourino@sescam.jccm.es (L.M.-A.); 4 Department of Internal Medicine, Hospital Nacional de Parapléjicos SESCAM, 45071 Toledo, Spain; miguelrc46@hotmail.com (M.R.-C.); irena1364@hotmail.com (I.J.-V.); gambarrutta@yahoo.es (C.G.-M.); 5 Proteomics Facility, Centro Nacional de Biotecnología (CNB), 28049 Madrid, Spain; fcorrales@cnb.csic.es; 6 Department of Rehabilitation, Hospital Nacional de Parapléjicos, SESCAM, 45071 Toledo, Spain; fgutierrezhenares@gmail.com (F.G.-H.); lamidolado@gmail.com (E.L.-D.); amgila@sescam.jccm.es (A.G.-A.)

**Keywords:** coronavirus, COVID-19, spinal cord injury, heparin

## Abstract

The COVID-19 pandemic represents an unprecedented global challenge in this century. COVID-19 is a viral respiratory infection, yet the clinical characteristics of this infection differ in spinal cord injury patients from those observed in the general population. Cough and asthenia are the most frequent symptoms in this population. Moreover, infected spinal cord injury patients rarely present complications that require admission to an Intensive Care Unit, in contrast to the general population. Thus, there is a clear need to understand how COVID-19 affects spinal cord injury patients from a molecular perspective. Here, we employed an -omics strategy in order to identify variations in protein abundance in spinal cord injury patients with and without COVID-19. After a quantitative differential analysis using isobaric tags and mass spectrometry and a verification phase, we have found differences mainly related to coagulation and platelet activation. Our results suggest a key role of heparin in the response of spinal cord injury patients to COVID-19 infection, showing a significant correlation between these proteins and heparin dose. Although the number of patients is limited, these data may shed light on new therapeutic options to improve the management these patients and, possibly, those of the general population as well.

## 1. Introduction

The recent Coronavirus outbreak (COVID-19) that emerged in Wuhan in December 2019, the capital of China’s Hubei province [1,2,3], has provoked a public health crisis of global proportions [4]. This viral pandemic is caused by the severe acute respiratory syndrome coronavirus 2 (SARS-CoV-2), which has spread intense illness, death and fear across the planet, well beyond that known in our times [5]. The specific manifestations reported include cough, fever and shortness of breath. Moreover, the demographic and clinical factors associated with a more aggressive COVID-19 phenotype include male gender, age over 60 years, and the presence of previous comorbidities like hypertension, diabetes mellitus, obesity, cardiac ischemic disease, lung disease and immunosuppression [6].

Spinal cord injury (SCI) is the result of temporary or permanent damage provoked by traumatic or non-traumatic causes at any level of the spinal cord. SCI has a significant effect on the individual’s physical and psychosocial well-being [7], and it is associated with considerable healthcare costs, morbidity and mortality, especially at advanced stages [8]. Furthermore, SCI produces multiple and quite specific neurological and systemic complications, which drastically reduce the individual’s life expectancy and quality of life (QoL: 8). As such, individuals with SCI clearly constitute a population that is particularly vulnerable to COVID-19, mostly due to the respiratory failure they experience as a result of thoracoabdominal muscle weakness [9] but also due to the systemic immunosuppression caused by their lesions [10,11]. After SCI, an autoimmune response provokes the suppression of the immune system in patients, principally driven by excessive glucocorticoid release as a results of stimulation of the hypothalamus-pituitary-adrenal (HPA) axis, in addition to noradrenergic overactivation [12]. It is important to note that patients with cervical or high-thoracic SCI suffer more notable immunosuppression and respiratory failure than those with lumbar injury, and for this reason, pneumonia is the main cause of mortality in these patients [13]. Given that COVID-19 is a viral respiratory infection, there is clearly an urgent need to know how this virus affects SCI patients and if they show fundamental differences relative to COVID 19 patients without SCI.

**Figure 1:**
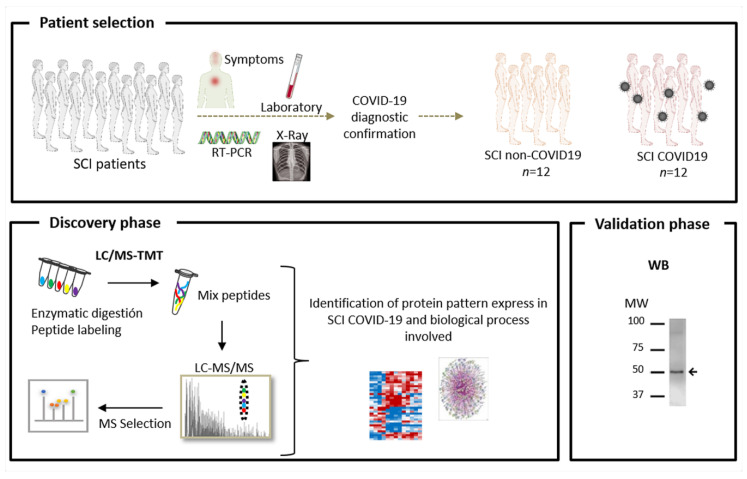
Schematic representation of the workflow. Plasma samples were collected from patients with spinal cord injury (SCI) with and without confirmed COVID-19 infection. The experimental design consists of a discovery phase using TMT labelling, followed by LC-MS/MS and a confirmation phase employing Western blotting (WB).

While enormous efforts have been expended to better understand the mechanisms of action of this virus in order to impede its transmission, as well as to improve its diagnosis and treatment, SCI patients may require more precise and specific attention. In this study, we show how applying an omics strategy helps identify variations in protein abundance in SCI patients with COVID 19 in an unbiased manner (Figure 1). For the first time, we define a specific protein signature in SCI-COVID 19 patients that probably reflects important biological process involved in the pathology of this disease. As a result, new therapeutic options have been discovered that might help improve the clinical management of these patients.

The results obtained here highlight the role of heparin in the specific response of SCI patients to COVID-19 infection, showing a significant correlation with proteins implied in coagulation/platelet activation.

## 2. Materials and Methods

### 2.1. Study Population and Sample Preparation

The patients enrolled inthis study had been previously admitted for clinical care at the Hospital Nacional de Parapléjicos in Toledo (Spain), and they were classified in the Hospital’s department of Internal Medicine. Clinical data was collected for each patient, which included their age, sex, heparin dose, neurological level and severity, with the last two variables assessed in accordance with the International Standards for Neurological Classification of SCI [14] (Table 1). Cases of COVID-19 were confirmed by reverse transcription polymerase chain reaction (RT-PCR), and based on their symptoms, laboratory tests and chest X-rays. 

For the analyses performed here, 7 mL of blood was withdrawn from each patient and introduced into EDTA-prepared collection tubes (Venoject, Terumo Europe). These samples were then immediately taken to the laboratory to prevent sample degradation (less than 2 h), they were centrifuged at 3500× *g* for 10 min at 4 °C, and the resulting plasma was subjected to chemical viral inactivation with acetone. Finally, the samples were aliquoted into batches (500 µL) and stored at −80 °C until the proteomic analysis was performed. All work procedures, risk assessments and safety measures were approved by the Biosafety Committee at the Hospital Nacional de Parapléjicos. 

Last, but not least, and in order to avoid bias and to ensure the patients’ data confidentiality, all documents were handled after removing personal or identifying data.

The experimental strategy was divided into two steps: (1) a discovery phase carried out on 16 plasma samples using Tandem Mass Tags (TMT) followed by LC-MS/MS and (2) a verification phase performed on 24 plasma samples by Western blotting and MS.

### 2.2. Quantitative Proteomic Analysis

#### 2.2.1. Protein Digestion and Isobaric Labelling

For the quantitative differential LC-MS/MS analysis using isobaric tags (TMT 10-plex), about 300 µg of total protein was digested using the filter-aided sample preparation (FASP) protocol described previously [15], with minor modifications. Samples were denatured by boiling for 5 min in the presence of 1% Sodium Dodecyl Sulfate (SDS) and 50 mM dithiothreitol (DTT), diluted in 7 M urea in 0.1 M Tris-HCl (pH 8.5: UA buffer) and loaded onto 10 kDa centrifugal filter devices (NanoSep 10k Omega, Pall Life Sciences, Port Washington, NY, USA). Samples were alkylated for 45 min at 37 °C in the dark in 50 mM IAA (iodoacetamide) in UA buffer. The excess alkylating reagent was eliminated by washing two times with UA buffer and two more times with 100 mM ammonium bicarbonate. The proteins were then digested overnight at 37 °C with modified trypsin (30:1 protein:trypsin (*w*/*w*) in 100 mM ammonium bicarbonate), and the resulting peptides were twice eluted by centrifugation with 100 mM ammonium bicarbonate and 0.5 M sodium chloride. Trifluoroacetic acid (TFA) was added to a final concentration of 1%, and the peptides were desalted onto C18 Oasis-HLB cartridges and dried for further analysis.

For stable isobaric labelling, the tryptic peptides obtained were dissolved in 100 mM Triethylammonium bicarbonate (TEAB) buffer and the peptide concentration was determined by measuring the amide bonds with the Direct Detect system (Merck Millipore, Burlington, MA, USA). Equal amounts of each peptide sample were labelled using the 10-plex TMT Reagents (Thermo Fisher, Waltham, MA, USA) according to the manufacturer’s instructions. The peptides were labelled with the TMT reagents previously reconstituted in 42 μL of acetonitrile (ACN), and after incubation at room temperature for 2 h, the reaction was stopped by adding 0.5% TFA for 30 min. The samples were concentrated in a Speed Vac, desalted onto C18 Oasis-HLB cartridges and dried for further analysis.

#### 2.2.2. Protein Identification and Quantitation

Labelled peptides were analyzed by LC-MS/MS using a C-18 reversed phase nano-column (75 µm I.D. × 50 cm, 2 µm particle size, Acclaim PepMap RSLC, 100 C18: Thermo Fisher Scientific, Waltham, MA, USA) and a continuous ACN gradient consisting of: 0–30% B for 360 min, 50–90% B in 3 min (A = 0.1% formic acid; B = 90% ACN, 0.1% formic acid, FA). A flow rate of 200 nL/min was used to elute peptides from the nano-column to an emitter nanospray needle for real time ionization and peptide fragmentation on an Orbitrap Fusion mass spectrometer (Thermo Fisher, Waltham, MA, USA). An enhanced FT-resolution spectrum (resolution = 70,000) and the MS/MS spectra from the Nth most intense parent ions were analyzed in the chromatography run. Dynamic exclusion was set at 40 s. For peptide identification, all the spectra were analyzed with Proteome Discoverer (version 2.1.0.81, Thermo Fisher Scientific, Waltham, MA, USA) using SEQUEST-HT (Thermo Fisher Scientific, Waltham, MA, USA).

The Uniprot database that contains all the sequences from human and contaminants was searched, selecting the parameters: trypsin digestion with 2 maximum missed cleavage sites; precursor and fragment mass tolerances of 2 Da and 0.02 Da, respectively; TMT modifications at N-terminal and Lys residues as fixed modifications; and methionine oxidation, carbamidomethyl cysteine and MMTS modified-cysteine as dynamic modifications (discovery phase). Peptide identification was performed using the probability ratio method [16], and the false discovery rate (FDR) was calculated using inverted databases and the refined method [17], with an additional filter for precursor mass tolerance of 15 ppm. The peptides identified had a FDR ≤ 1% and only these peptides were used to quantify the relative abundance of each protein from reporter ion intensities. For statistical analysis of the quantitative data, the previously described weighted spectrum, peptide and protein (WSPP) statistical model was used [18]. In this model, the protein log2-ratios are expressed as standardized variables, i.e., in units of standard deviation according to their estimated variances (Zq values).

### 2.3. Functional Group Analysis

To examine the function of the proteins identified, the list of 33 proteins that varied significantly was implemented in the on-line David Bioinformatics Resources 6.8 (NIH) software for their functional analyses [19]. Functional annotation clustering was performed to avoid redundancy of enriched categories and pathways. 

### 2.4. Western Blotting

Equal amounts of protein samples (25 µg) obtained from SCI patients with and without COVID-19 were resolved by 10% SDS–polyacrylamide gel electrophoresis in a Bio-Rad Miniprotean II electrophoresis cell run at a constant current of 25 mA/gel. After electrophoresis, the proteins were transferred to a nitrocellulose membrane under a constant voltage of 20 V for 30 min, and the membranes were stained with Ponceau S to guarantee an equal amount of protein was loaded for each patient. Subsequently, the membranes were blocked for 1 h with Phosphate-buffered saline-Tween 20 (PBS-T) containing 7.5% non-fat dry milk and incubated overnight with the primary antibody in PBS-T with 5% non-fat dry milk. The primary antibodies used were a rabbit polyclonal antisera against fibrinogen alpha chain (FIBA, 1/5000, Abcam ab92572), fibrinogen beta chain (FIBB, 1/1000, Abcam ab193932), fibrinogen gamma chain (FIBG, 1/500, Abcam ab92481) and a mouse monoclonal antibody against haptoglobin (HPT, diluted 1/100: Abcam, Ref. ab13429). After washing, the membranes were incubated with a specific HRP-conjugated secondary antibody in PBS-T containing 5% non-fat dry milk and antibody binding was detected by enhanced chemiluminescence (ECL: GE Healthcare, Little Chalfont, UK), according to the manufacturers’ instructions. Densitometry was performed with the ImageQuantTL software (GE Healthcare, Little Chalfont, UK).

### 2.5. Statistics

Normality was assessed with the Kolmogorov–Smirnov test, and the data are expressed as the means ± standard deviation (SD) or percentages. Two-tailed Student *t*-tests were employed to calculate the differences between groups. A two-tailed Pearson’s correlation coefficient was calculated to analyze the association between two variables using SPSS 15.0 for Windows (SPSS Inc. Chicago, IL, USA). Statistical significance was accepted at: * *p* < 0.05, ** *p* < 0.01, *** *p* < 0.001.

### 2.6. Study Approval

The study was approved by the Ethics Committee of the ComplejoHospitalario de Toledo (Spain), and it was carried out according to the principles of the Helsinki Declaration. All patients signed their written informed consent prior to their inclusion. To avoid any bias and to ensure patient confidentiality, all documents were handled after removing personal or identifying data. 

## 3. Results

**Figure 2:**
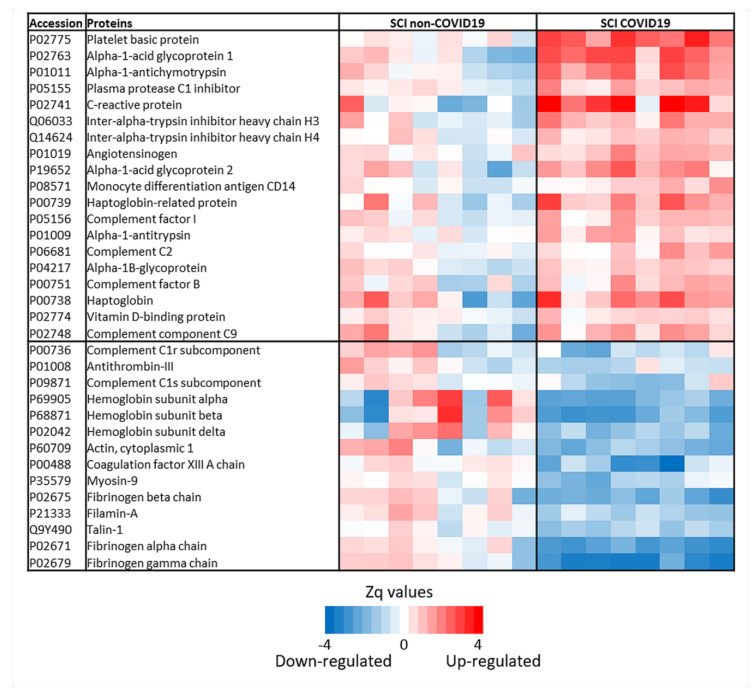
Differentially expressed proteins identified by Tandem Mass Tag labelling. Heatmap representing the changes in protein abundance between the groups. Zq (standardized log2 ratio) values are represented in a color scale (red means up-regulated and blue down-regulated). The protein name and UniProt accession codes are shown. Two-tailed Student *t*-tests were employed to calculate the differences between groups.

In this work, we have applied a proteomic approach to gain a deeper understanding of the molecular mechanisms associated with COVID-19 infections in patients with SCI. Using this strategy, we identified a total of 500 proteins of which 207 were detected in the 16 samples analyzed in the discovery phase. Of these, 33 proteins were differentially expressed in SCI patients, with 20 upregulated and 13 downregulated. The quantitative variability of the differentially expressed proteins found in SCI patients with COVID-19 was represented in a heatmap (Figure 2), in which patients with and without COVID-19 were perfectly separated. This heatmap also shows that the non-COVID group is more heterogeneous, presenting more between subject variability.

The function of the proteins altered was evaluated using DAVID v6.8. The molecular functions and biological processes were explored using the functional annotation tool to generate clusters of overrepresented Gene Ontology (GO) terms, identifying 3 significant clusters. Of these, the most significant and abundant cluster was formed by 21 GO terms and 9 proteins that are related to blood coagulation and platelet activation (Table 2). To verify these results, a group in whichwe included an independent cohort of subjects was used. Firstly, an additional TMT experiment was performed to confirm the trend of the differential proteins. Besides, 4 of the proteins belonging to these significant clusters were analyzed in Western blots, in order to use an orthogonal technique, different from mass spectrometry.

**Figure 3:**
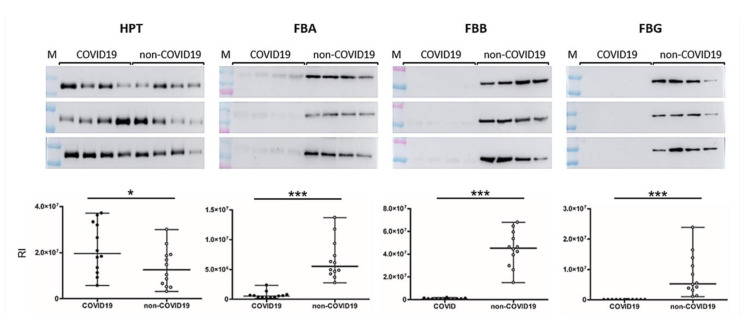
Validation of the differentially expressed proteins found in discovery phase by Western blotting. Box and Whiskers plots represent differentially expressed proteins. COVID-19 patients are situated on the left side and non-COVID-19 patients on the right side of each membrane. Two-tailed Student *t*-tests were employed to calculate the differences between groups. * and *** showed statistical significance, * *p* < 0.05, *** *p* < 0.001. HPT, haptoglobin; FIBA, fibrinogen alpha chain; FIBB, fibrinogen beta chain; FIBG, fibrinogen gamma chain; M: molecular weight marker; RI, relative intensity.

**Figure 4:**
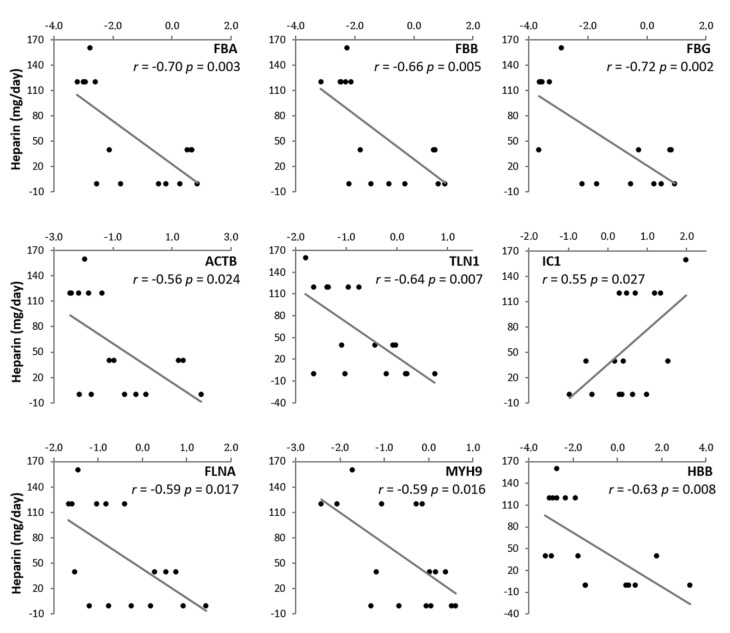
Correlations between coagulation proteins and heparin dose. Y-axis represents heparin dose while x-axis represents Zq values of the different proteins. Two-tailed Pearson’s correlation coefficient was calculated to analyze the association between two variables. Pearson coefficient and *p*-value are shown.

In Western blots, HPT was clearly more abundant in SCI COVID-19 patients relative to non-COVID-19 patients (*p* = 0.049). By contrast, both FIBA, FIBB and FIBG were downregulated in SCI COVID-19 patients (*p* < 0.001, Figure 3). Correlations were established to determine if the amount of heparin significantly affected the levels of coagulation/platelet activation proteins, which proved to be the case (Figure 4). Plasma protease C1 inhibitor was upregulated in COVID-19 patients, displaying a positive correlation, while FIBA, talin 1, filamin A, myosin heavy chain 9 and the beta subunit of hemoglobin, displayed negative correlations. 

## 4. Discussion

Individuals that suffer SCI experience a myriad of physiological changes, such as respiratory dysfunction, temperature dysregulation or impaired cough, all of which may increase their risk of morbidity and may delay the diagnosis of COVID-19, not least because they exhibit fewer symptoms than the general population [20]. Nevertheless, although their susceptibility is higher, as is the MEWS (Modified Early Warning Score) they present, the clinical evolution of COVID-19 in these patients is not as severe as expected [21]. Taking this information into account and given the limited information available regarding the unusual course of COVID-19 in SCI patients [22], it is important to define novel biomarkers that might allow us to identify the biological processes involved in the pathological events in these patients. Only by identifying these features can new therapeutic options be discovered to improve the clinical management of these specific patients but also, to elucidate the differences underlying the better outcome in these patients than in patients without SCI. To the best of our knowledge, this is the first proteomic study to be carried out on the plasma of SCI COVID-19 patients, through which we have been able to define specific patterns of protein expression in these patients. 

In this study, we applied a multi-proteomic approach to analyze plasma samples from two independent SCI cohorts, one that included patients infected by COVID-19 and the other, control uninfected SCI patients. In order to maximize safety, the plasma was subjected to chemical viral inactivation with acetone, and protein extracts were used in all the experimental steps, even though it has been proposed that only 1% of blood samples from COVID-19 patients have a positive viral load test [23]. Through these proteomics analyses, and after a verification phase where we included new SCI patients with and without COVID-19, we found 33 altered proteins, 12 of which belong to the 3 significant clusters according to GO terms.

The most abundant cluster is related to coagulation and platelet activation, and it is formed by fibrinogens (alpha, beta and gamma chains), beta actin, talin 1, plasma protease C1 inhibitor, filamin A, myosin heavy chain 9 and the beta subunit of hemoglobin. Of these, the fibrinogens, talin and actin are directly implicated in platelet activation. These proteins play an essential role in thrombus formation, and actin and talin are indispensable for the formation of a stable fibrinogen-integrin αIIbβ3-actin cytoskeleton complex [24,25,26]. In the same way, filamin A is essential for platelet aggregation [27,28], and myosin heavy chain 9-related disorders are associated with a low number of platelets [29,30]. The role of plasma protease C1 inhibitor in the regulation of fibrinolysis is also interesting, as it inhibits the conversion of soluble fibrinogen into insoluble strands of fibrin [31].

Fibrinogen is the soluble blood precursor of fibrin, and it contributes to elevated plasma viscosity and the risk of fibrin clot formation. Moreover, it plays important roles in fibrinolysis, cellular and matrix interactions, inflammation, and wound healing [32]. Fibrinogen up-regulation has been detected in severe COVID-19 cases, highlighting the importance of coagulation in this infection [33]. Moreover, a similar change was also described following SARS infection, suggesting an important role in the pathogenesis of this virus [34]. Nevertheless, and in contrast to the population without SCI, there was a remarkable downregulation of fibrinogen and other pro-thrombotic proteins in SCICOVID-19 patients. The contrasting levels found in this population could explain why the clinical evolution of COVID-19 infection in SCI patients was not as pronounced as expected. Indeed, the SCICOVID-19 population does not develop thrombosis, which has been associated with COVID-19 infection in other individuals [35,36].

We should also pay attention to hemoglobin and HPT function in this pathology. HPT is known as an acute phase protein in serum [37], and it was described in SARS-CoV, explaining the possibly inhibition of neutrophil respiratory burst activity and its angiogenic role in lung tissue repair [38]. In addition, a proteomic study of cerebrospinal fluid from SCI patients identified increased levels of HPT in patients with incomplete injury (AIS grade A or D), and a decrease 15–60 days post injury in both complete and incomplete cases [39]. However, the patients studied here comply withdifferent AIS grades, and the number of days post injury varies from 19 days to 1 year in both groups. Hence, the changes in HPT levels found here appear not to depend on injury severity, but rather, they depend on virus infection. In addition to its angiogenic factor and anti-inflammatory properties, HPT forms a complex with hemoglobin, and it plays an important role in combating oxidative stress [40,41]. Indeed, when hemoglobin levels have been assessed in COVID-19 patients without SCI, lower levels of this protein were associated with poorer outcomes [42,43]. Nevertheless, patients with low hemoglobin recovered well from the disease.

Although further studies will be needed, these results provide information to improve understanding the specific response to COVID-19 infection in SCI patients. While physiological characteristics linked to SCI should be taken into account, differential heparin levels may be one of the keys to this response. The data show astatistical correlation between all the proteins implied in coagulation/platelet activation and the heparin dose administered. It is important to highlight here that SCI patients with COVID-19 receive prophylactic heparin to avoid thrombotic and embolic complications typical of the SCI. This dose is increased from 40 mg/24 h, the normal prophylactic dose in SCI patients, to 60–80 mg/12 h in SCI COVID-19 infected patients from the moment of diagnosis. Importantly, the dose of 40 mg/24 h is also the most common dose that non-SCI COVID patients receive in a prophylactic way, according to general recommendations. There are several studies that propose heparin treatment for COVID-19 patients [44,45]. Recently, it has been suggested that heparin might have therapeutic potential against SARS-CoV-2 infection as a competitive inhibitor [45,46,47,48]. Our study supports the used of heparin as treatment of choice in COVID-19, especially in SCI patients. Additionally, this higher heparin dose shows the efficacy and safety in this SCI population, and it should be taken as a starting point for future studies.

This study has several limitations that should be taken into account. First, these analyses have been performed in a small cohort of patients so greatest studies should be made to confirm the clinical relevance of this heparin dose. Last but not least, it is important to note that only a small proportion of the population suffer a SCI, and this is one of the reasons why our cohort is small. Secondly, this study should ideally include patients without SCI, both with and without COVID-19 infection, in order to ensure that this treatment is effective for the general population. Nevertheless, our results point out the importance of the heparin treatment in SCI patients with COVID-19 infections and may open the door to general thromboprophylaxis from the moment of COVID-19 diagnosis.

## Figures and Tables

**Table 1:** Clinical characteristics of the subjects used in the study. ASIA, American Spinal Injury Association; M/F, male/female; NA, not applicable.

		Non COVID	COVID	*p*-Value
**Age (years)**		52.7 ± 17.1	61.1 ± 14.4	0.22
**Gender (%M/F)**		72/28	73/27	0.86
**ASIA** **Impairment scale (%)**	**A**	54	45	NA
	**B**	8	9	NA
	**C**	23	9	NA
	**D**	15	18	NA
**Heparin (%)**		46	82	0.15
**Heparin dose (mg/day)**		40	98	0.03

**Table 2:** Results of the functional annotation clustering showing the *p*-value of each term and the fold change (FC).

	Term	*t*-test	FC	Proteins
Cluster 1 Enrichment Score: 1.69	platelet aggregation	0.000	7.3	fibrinogen alpha chain (FGA); fibrinogen beta chain (FGB); fibrinogen gamma chain (FGG); plasma protease C1 inhibitor (IC1); filamin A (FLNA); myosin heavy chain 9 (MYH9); actin beta (ACTB); hemoglobin subunit beta (HBB); talin 1 (TLN1)
	positive regulation of substrate adhesion-dependent cell spreading	0.004	10	
	cellular protein complex assembly	0.016	13	
	positive regulation of vasoconstriction	0.016	13	
	positive regulation of peptide hormone secretion	0.016	13	
	negative regulation of endothelial cell apoptotic process	0.016	13	
	negative regulation of extrinsic apoptotic signaling pathway via death domain receptors	0.016	13	
	Platelet activation	0.018	4.1	
	protein binding, bridging	0.025	10	
	positive regulation of heterotypic cell-cell adhesion	0.030	9.6	
	positive regulation of exocytosis	0.030	9.6	
	positive regulation of protein secretion	0.030	9.6	
	response to calcium ion	0.030	9.6	
	protein polymerization	0.030	9.6	
	blood coagulation, fibrin clot formation	0.030	9.6	
	plasminogen activation	0.048	7.7	
	cell adhesion molecule binding	0.058	7	
	fibrinolysis	0.080	3.7	
	fibrinogen complex	0.087	5.7	
	platelet alpha granule	0.087	5.7	
	positive regulation of ERK1 and ERK2 cascade	0.091	5.5	
Cluster 2 Enrichment Score: 1.55	haptoglobin-hemoglobin complex	0.015	13	Haptoglobin (HP); hemoglobin subunit beta (HBB); hemoglobin subunit alpha 1 (HBA1)
	positive regulation of cell death	0.016	13	
	response to hydrogen peroxide	0.030	9.6	
	cellular oxidant detoxification	0.091	5.5	
Cluster 3 Enrichment Score: 1.38	oxygen transporter activity	0.025	10	Hemoglobin subunit delta (HBD); hemoglobin subunit beta (HBB); hemoglobin subunit alpha 1 (HBA1)
	hemoglobin complex	0.028	9.9	
	oxygen transport	0.030	9.6	
	oxygen binding	0.041	8.4	
	iron ion binding	0.078	6	
	heme binding	0.078	6	
